# Pet-Assisted Therapy for Delirium and Agitation in Hospitalized Patients with Neurocognitive Impairment: A Review of Literature

**DOI:** 10.3390/geriatrics6040096

**Published:** 2021-10-01

**Authors:** Abu Baker Sheikh, Nismat Javed, Katarina Leyba, Ali Hamza Khair, Zainab Ijaz, Aimen Asim Dar, Hamza Hanif, Asif Farooq, Rahul Shekhar

**Affiliations:** 1 Department of Internal Medicine, University of New Mexico Health Sciences Center, Albuquerque, NM 87131, USA; katarinaleyba@gmail.com (K.L.); rahul547843@gmail.com (R.S.); 2 Shifa College of Medicine, Shifa Tameer-e-Millat University, Islamabad 44000, Pakistan; nismatjaved@gmail.com (N.J.); sahamza96@gmail.com (A.H.K.); aimen_dar97@hotmail.com (A.A.D.); 3 Department of Psychiatry, William Beaumont Hospital, Royal Oak, MI 48073, USA; zijaz@ualberta.ca; 4 Department of General Surgery, University of New Mexico Health Sciences Center, Albuquerque, NM 87131, USA; hhanif@salud.unm.edu; 5 Department of Family and Community Medicine, Texas Tech Health Sciences Center, Lubbock, TX 79430, USA; afarooq76@gmail.com

**Keywords:** agitation, delirium, pet-assisted intervention, polypharmacy

## Abstract

Delirium leading to agitation is a common issue in elderly people and patients with underlying neurocognitive impairment. Despite use of medications to treat agitation, polypharmacy is a major concern and might lead to multiple side effects in this patient population. Therefore, it is imperative to investigate non-pharmacological methods that can provide solutions to the problem. The objective of this review was to evaluate the impact of pet-assisted therapy on elderly patients, with a major focus on agitation and delirium. For the purposes of this study, a scoping review was performed using PubMed, Google Scholar, and ClinicalTrials. We reviewed literature from 1980 to 2021. Out of the 31 studies reviewed, 14 commented on agitation with respect to pet-assisted interventions. Of these, eight studies (57%) reported a statistically significant reduction in agitation and/or delirium in patients who were exposed to pet therapy. Pet-assisted therapy can improve the standardized care in hospital-based settings for patients with neurocognitive impairment because of better companionship, reduced agitation and mood disorders, and better stability of hemodynamic status. These interventions can pave the way for better patient and hospital satisfaction.

## 1. Introduction

Delirium is an acute confusional state illustrated by altered conscious levels along with a reduced ability to focus, sustain, or shift attention. Delirium mostly develops acutely and follows a waxing and waning pattern [1]. It is usually characterized by psychomotor and autonomic hyperactivity that manifests as agitation and hallucinations [1]. Some of the important causes include severe or chronic illness, metabolic imbalances, drugs, infections, or surgery. Additionally, other risk factors include any condition that results in a prolonged hospital stay, being a resident of a nursing home, and preexisting neurocognitive disorders such as dementia.

In addition to pharmacologic interventions (antipsychotics, benzodiazepines, and cholinesterase inhibitors), many non-pharmacologic interventions are also commonly employed, which range from reducing modifiable risk factors to unorthodox methods such as aromatherapy, music intervention, massage, and multi-sensory stimulation [2]. One such non-pharmacological treatment is animal-assisted therapy (AAT), which entails the introduction of animals into patient settings in order to improve patient agitation, anxiety, and mood [3].

Very early medical texts have already described the therapeutic effects of animal companionship. Hippocrates and Galen were early proponents, suggesting horse-riding as a cure to insomnia and to prevent disease [4]. The structured use of animals as an aid in treating mental and physical health disorders dates back to 1792 [4].

Indications for the use of animal-assisted therapy in the literature vary widely, but mostly focus on diseases with neurological or psychiatric components [3]. The elderly population has been especially studied in preference to the pediatric and adult population [3]. The outcomes of the studies, while generally positive, have proven difficult to quantify as a result of vastly different patient settings, length of animal encounters parameters measured, and the indication for the initiation of AAT. While most of these therapies have been usually studied in the setting of dementia, outcomes suggest they may also have a place in treating acute confusional states and agitation.

The objective of this review was to evaluate the impact of pet-assisted therapy on elderly patients, with influence on agitation and delirium as the primary outcome. The secondary outcome highlighted cognition, quality of life, physical functioning, vitals, and depressive/mood disorders.

## 2. Methods

For the purposes of this study, a scoping review was performed. PubMed, Google Scholar, and ClinicalTrials.gov were used for the retrieval of studies required for the review. We reviewed literature from 1980 to 2021. The keywords used in the review were “dementia”, “agitation”, “delirium”, “pet assisted therapy”, “cognition”, “elderly”, and “psychiatry”.

### 2.1. Studies Reviewed for Primary and Secondary Outcomes

This scoping review included 31 articles that studied the impact of pet-assisted therapy on the health of elderly patients, including 16 interventional studies, 9 randomized controlled trials, 4 observational studies, and 2 studies with a nonequivalent control group pretest−posttest design. The primary outcome analyzed in this review was the effect of pets or pet robots on agitation or delirium. The secondary outcome analyzed in this review was the effect of pets or pet robots on cognition, depression and loneliness, quality of life, blood pressure, physical functioning, and activities of daily living (ADL).

### 2.2. Population Studied

The population of interest in this study is older adults, especially those with dementia. The studies analyzed in this review all examined the effect of pet therapy on older patients, but the inclusion criteria for this population varied among studies. Of these studies, 15 utilized populations of older patients residing at long-term care facilities, 11 focused on the population of elderly adults in nursing homes, and 3 focused on elderly patients in inpatient units. Eight studies used an age criterion of >65, two studies used an age criteria of >60, one study used an age criteria of >55, and one study used an age criteria of 80 to 90 years of age. A further 24 studies narrowed their study populations by only including patients with dementia.

### 2.3. Intervention

Of the 31 studies included in this analysis, 12 (39%) studied living pets as a form of therapy. The majority of studies utilized dogs or cats, but two studies also included fish, and one included rabbits, birds, and horses as therapy animals as well. In contrast, 14 studies (45%) investigated pet robots as a form of therapy. Interventions ranged from individual therapy sessions to group therapy session to cohabitating with an animal.

## 3. Review

Summarized data from all articles analyzed in this review are reported in Table 1 and Table 2 [5,6,7,8,9,10,11,12,13,14,15,16,17,18,19,20,21,22,23,24,25,26,27,28,29,30,31,32,33,34,35].

### 3.1. Primary Outcomes

Fourteen out of thirty-one studies in this analysis investigated the primary outcomes. Of these, eight studies (57%) reported a statistically significant reduction in agitation and/or delirium in patients who were exposed to pet therapy, and six (36%) reported no statistically significant difference. The majority of the studies included in the review reported decreased agitation and aggression in patients [5,6,7,8,9,10,11,12,13,14,15,16,17,18]. There are certain characteristics of the animal-assisted interventions that might be responsible for the decrease in agitation. The pets, and their actions and voices, overall, have a calming influence over the subjects and might even help in emotional expression in these cases [36]. These interventions remind patients of the comfortable environment at home [36]. Patients could also have a false sense of distraction from their current environment, which could assist the healthcare team in providing optimal care [36].

With regard to pet assisted therapy, dogs were the most frequently employed animals given their training potential and social nature [37]. However, numerous other animals, including robotic pets, have also been utilized. The comfort level might be more pronounced if the patient’s preference is taken into account and the choice of pets is not restricted [36]. The positive impact might also be increased in patients who are not visited by family members at all, either due to time constraints or geographical limitations [36,37]. The result is that an equally important facet of patient care in this population, namely companionship, begins to develop, and there is less sense of being dependent on an individual [37].

There are also rare cases where agitation is increased instead of a desirable effect [38].

This could possibly be explained by the idea of hyperactive delirium that might have been present in these patients. A paper published in 1985 presented a possible theory. Hyperactive delirium, seen postoperatively, is associated with levels of beta-endorphin and cortisol [39]. Endorphins are naturally increased when a pleasing stimulus, for example, a pet, comes into view. Therefore, this cycle of a negative impact could be initiated [39].

Two systematic reviews and one meta-analysis also discussed the impact of animal-assisted intervention on agitation [40,41,42]. However, the reviews were focused on a few articles and excluded a number of articles with important findings pertaining to agitation. Hughes et al. included Majić et al., Richeson, Sellers et al., and Nordgren and Engström, but the review did not include other important articles and did not summarize a focus on agitation as done for other aspects [5,6,11,14,40]. Similarly, Park et al. did not include Sellers et al., Richeson, or Nordgren and Engström, who noted a significant reduction and exacerbation in agitative behaviors in these patients [6,11,14,41]. A similar observation was made by Bert et al. [42]. Therefore, the findings of these reviews have to be validated in a larger setting with an appropriate intervention.

### 3.2. Secondary Outcomes

An improvement in cognition was noted in 22% of the articles reviewed. A significant decrease in depression was also reported by Friedmann et al. in the PAL group (*p* = 0.013) and by Abdi et al. in 11/33 studies [16,43]. Furthermore, owing to reduced mood disorders, cognition in these patients can improve as observed by a few studies [5,12,14,23,27,29]. Improvement in loneliness were also reported by 16% of the studies. Animal-assisted interventions were also responsible for a reduction in anxiety, sadness, and irritability [27,29]. The possible explanation for this could be that animal-based interactions might ease suffering and build neuronal networks not targeted by pharmacological methods [44]. Furthermore, because these patients usually live in a very lonely environment, pets could help in potentially humanizing the wards to debunk some of the negative opinions that have arisen over the years [44]. Once patients engage more in the rehabilitating efforts done by the healthcare teams as a result of these interventions, quality of life might be improved, as reported by Bert et al. [42].

Animal assisted interventions might also improve physical functioning and activities of daily living, as witnessed by Friedmann et al. and Cherniack and Cherniack in the PAL group compared with the reminiscing group (*p* = 0.306 vs. 0.072) [16,45]. This might be due to patient curiosity about the actions and interactions of the pets [36]. Other reasons also include distraction from chronic pain and a sense of responsibility for the pets in question [46].

The improvement in physical functioning is not only limited to depression and cognition; the overall health of the patients might improve as well, for example, a reduction in systolic blood pressure was reported by a few articles [16,19]. All these changes can improve the overall quality of life and decrease the length of stay in hospitals [16].

### 3.3. Pet-Assisted Interventions in Hospital Setting

Hospitals are high-risk areas, thus introducing animals has to be carefully considered. There are a few risks associated with the intervention, such as infections, allergies and animal accidents [47,48,49,50]. The odds of contracting zoonosis and MRSA are higher [51,52]. Additionally, the more chronic patients would require these interventions the most. Patients with chronic illnesses include immunocompromised or malnourished categories. However, these categories were excluded from a majority of the reviews due to safety concerns. Therefore, there is a need to personalize these interventions in a way that benefits all categories. Perhaps the answer to this issue lies in using technology to invent robotic pets for this subset of patients.

Polypharmacy is a concern in this patient population, and conventional therapies that include drugs such as antipsychotics and benzodiazepines that have considerable side effects. Patients with cognitive impairment also frequently require assistance at home especially in regards to drug administration. These factors together add considerable cost on top of the original patient treatment, and is an area where pet assisted therapy may help. Thus, this is another area where pet-assisted therapy may be especially useful because the approach could be used to reduce resources utilized in a hospital setting in arranging home care, the administration of medication, and overall patient load so that optimal healthcare can be ensured. Additionally, there would be minimal risk of relapse because less medications would be used, reducing the need for longer stays to taper medications. The length of stay of most patients with underlying neurocognitive impairment would decrease in a hospital-based setting should pet assisted interventions be implemented because of a sense of safety and companionship. This might help the patients in engaging in rehabilitation regimens better than before. The quality of stay would also improve in a hospital setting because less patients would have hypertensive disorders owing to abrupt changes in their hemodynamic status. Overall, the satisfaction rate in the hospital would increase, not just for the patients but also for the healthcare providers, because the sense of responsibility of helping patients to have a better life out of the hospital is somewhat fulfilled.

There were a few limitations to the review. Some of the articles included were small centered studies, and the limited data available regarding this field makes the generalization of results a dilemma. In many articles, the search was limited to English language-based databases provided a very narrow ground for exploring more cases. Inclusion and exclusion criteria were not clearly stated in a few cases, necessitating the need for assumption, which might have introduced bias. In a few cases, the data were very heterogeneous and made the comparison of data an uphill task. Lastly, some articles did not control confounding variables such as exercise, which could have had some influence on the results.

## 4. Conclusions

Pet-assisted therapy can improve the standardized care in hospital-based settings for patients with neurocognitive disorders because of better companionship, reduced agitation and mood disorders, and better stability of the hemodynamic status. These interventions can pave the way for better patient and hospital satisfaction. However, to truly evaluate the novel invention, randomized controlled trials should target these methods in a hospital-based environment.

## Figures and Tables

**Table 1:** Summary of all of the studies reviewed.

Article	Year	Number of Participants	Setting	Study Type	Criteria	Intervention
Majic et al. [5]	2013	75	● Nursing home	● Matched case-control trial	● **Inclusion criteria**: Had a sum score on the Mini-Mental State Examination (MMSE) <25 From the Dementia of the Diagnostic and Statistical Manual of Mental Disorders, Fourth Edition (DSM-IV), the duration of cognitive impairment was <6 months, and having clinically significant cognitive impairment ● **Exclusion criteria**: Schizophrenia, bipolar disorder, or terminal somatic illness	● AAT was additionally conducted for 10 weeks ● AAT once a week for up to 45 min; Day of the week and time of dog visits remained constant; the dog therapy guide was present, conversing with the patient and introducing the therapy dog
Nordgren and Engström [6]	2013	33	● Nursing homes	● Interventional study	● **Inclusion criteria**: Dementia and being a resident at the nursing home for at least four weeks ● **Exclusion criteria**: Being allergic to dogs, anxiety towards dogs, or aggression towards dogs	● 10 sessions in total ● The total time for the intervention varied between participants because each protocol was personalized ● The time for each session was 45–60 min, at a frequency of once or twice a week), and the ability to be trained (cognitive, physical, or psychosocial)
Liang et al. [7]	2017	30	● Day care lefts and patients diagnosed with dementia	● Randomized controlled trial	● **Inclusion criteria**: dementia diagnosed ● **Exclusion criteria**: non-English speaking, moved away, no diagnosis of dementia, care recipient passed away, or refusal to participate	● PARO assisted intervention in one group and the other group acted as the control ● Sessions at day care and home for 6 weeks ● Follow-up at 12 weeks
Olsen et al. [8]	2016	58	● Nursing home	● Randomized controlled trial	● **Inclusion criteria**: Aged ≥65 years, having dementia, and a cognitive deficit score of <25 on the Mini-Mental State Examination Test ● **Exclusion criteria**: Nursing home residents with fear of dogs or dog allergy	● 30-min session with dogs twice weekly for 12 weeks in groups of three to six participants
Nurenberg et al. [9]	2015	105	● Inpatient setting	● Randomized controlled trial	● **Inclusion criteria**: Inpatients, 18 to 65 years old, aggressive or repressed behavior, persistent social isolation, and difficulty engaging in discharge-related programs ● **Exclusion criteria**: impaired ambulation, cognitive impairments, or other medical factors that might be exacerbated or result in harm during animal contact	● Active interventions (ten 40- to 60-min weekly) group sessions, with groups of up to ten members ● The standard control group received no additional interventions beyond regular hospital treatment
Churchill et al. [10]	1999	Not specified	● Not specified	● Not specified	● Not specified	● Animal-assisted intervention during the difficult “sundown” time (17:00–17:30 p.m.) in three SCUs to examine the effect on residents with a history of agitated “sundowning” behavior
Richeson et al. [11]	2003	15	● Nursing homes	● Interventional study	● **Inclusion criteria**: reside in a nursing home in SCU, 60 years of age or older, have a Mini-Mental State Examination (MMSE) score of 15 or below, be diagnosed with dementia as recorded by a physician in the resident’s medical record, have at least three documented agitated behaviors (e.g., screaming, biting, and spitting) in the last two months as recorded in the resident’s medical record, have a past interest in animals (e.g., owning a pet) as reported by family members, have no known allergies to dogs, have no known fear or intense dislike of dogs, and need an intervention for agitation as identified by the therapeutic recreation director ● **Exclusion criteria**: Not specified	● AAT in quasi-experimental time-series design with three phases: baseline (A) prior to intervention, post-test (B) after the three-week intervention, and follow-up (C) three weeks after the intervention ended; participants served as their own control
Jøranson et al. [12]	2015	53	● Nursing home for patients with dementia or mild neurocognitive impairment	● Randomized, controlled trial	● **Inclusion criteria**: >65 years with a dementia diagnosis or who met the criteria for cognitive impairment (<25/30), residents showed an interest in PARO when it was demonstrated during recruitment ● **Exclusion criteria**: None	● Supervised group interaction with PARO or TAU; two sessions/week for 12 weeks
Libin and Cohen-Mansfield [13]	2004	9	● Nursing home for patients with dementia	● Interventional study	● **Inclusion and exclusion criteria**: Not specified	● Supervised one-on-one interaction with NeCoRo and toy cat; one session only
Sellers et al. [14]	2006	4	● Patients with dementia	● Observational study	● **Inclusion criteria**: Elders in the facility with a documented presence of agitated behaviors, and a documented diagnosis of dementia or Alzheimer’s disease regardless of level of severity; an interest in and affectionfor animals; no allergic reaction to canines; and similar levels of abilities ● **Exclusion criteria**: Not specified	● A canine was utilized in the study, 15 min interaction/week
Moyle et al. [15]	2017	415	● Long term care facilities for patients with dementia	● Randomized, controlled trial	● **Inclusion criteria**: aged ≥60 years and a documented diagnosis of dementia ● **Exclusion criteria**: respite care admission, dual diagnosis of a serious/persistent mental illness, terminal illness, and unremitting pain/distressing physical symptoms	● Free one-on-one interaction with PARO switched on, PARO switched off, or TAU; 3 sessions/week for 10 weeks
Friedmann et al. [16]	2014	40	● Assisted living facilities	● Randomized controlled trial	● **Inclusion criteria**: Mild to moderate cognitive impairment (MMSE >8 and <23), age >55 years, anticipated length of stay of at least 6 months, English speaking, and interest in dogs ● **Exclusion criteria**: allergies/fear of dogs, hospice care, or asthma	● Pet-assisted living (PAL) group (60- to 90-min sessions over 12 weeks): 22/40 ● Reminiscing group (skill building over 12 weeks): 18/40
Zisselman et al. [17]	1996	58	● Psychiatry unit	● Interventional study	● **Inclusion criteria**: All patients hospitalized at the Geriatric Psychiatry Unit and the Will Eye Hospital in Philadelphia between February and May 1994 ● **Exclusion criteria**: None	● Pet therapy intervention: 33/58 ● Exercise intervention (the units usual activity programming): 25/58
Gustafsson et al. [18]	2015	4	● Dementia care home in Sweden	● Interventional study	● **Inclusion criteria**: Two men aged 82–90 years ● **Exclusion criteria**: None	● Supervised one-on-one interaction with JustoCat/week for 7 weeks
Krause-Parello and Kolassa [19]	2016	28	● Community dwelling of older adults	● Cross-over, interventional study	● **Inclusion criteria**: From a convenience sample recruited from Caregiver Volunteers of Central New Jersey, enrolled in the Caregiver Canines Therapy Dog Program, reside in independent housing, and able to communicate in English ● **Exclusion criteria**: None	● Two visits to each participant’s home: one from a volunteer-handler canine team, and one from a volunteer with no canine
Menna et al. [20]	2015	50	● Daycare left	● Interventional study	● **Inclusion criteria**: Mild to moderate Alzheimer’s disease ● **Exclusion criteria**: Behavioral problems	● Animal-assisted therapy (AAT): 20/50 ● Reality orientation therapy (ROT): 20/50 ● Control: 10/50
Moretti et al. [21]	2011	21	● Nursing home	● Interventional study	● **Inclusion criteria**: Age >65 years, institutionalized at least 2 months, affected by a mental illness (Alzheimer’s vascular dementia, secondary dementia, mood disorders, or psychotic disorders) as per participant’s medical record based on ICD-10 ● **Exclusion criteria**: Deafness/blindness or inability to interact with staff	● Pet therapy group: 10/21 ● Control group: 11/21
Petersen et al. [22]	2017	61	● Inpatient settings	● Interventional study	● **Inclusion criteria**: Diagnosed with mild to moderate dementia and age >65 years ● **Exclusion criteria**: Pre-existing psychiatric diagnosis, or unable to participate in programming due to physical limitations	● Treatment group: effect of the PARO robotic pet in treating dementia-related symptoms: 35/61 ● Control group: effect of standardized activity programs on dementia-related symptoms: 26/61
Song et al. [23]	2009	32	● Not specified	● Nonequivalent control group pretest-posttest design	● **Inclusion criteria**: Age >65 years, MMSE-K 10–24 points, no psychiatric history, no speech or hearing problems, and no organic brain lesions ● **Exclusion criteria**: Family members or participation in similar programs	● Robotic group: 17/32 ● Control group: 15/32
Sung et al. [24]	2015	16 enrolled; 12 completed the study	● Residential care facility	● Interventional study	● **Inclusion criteria**: Age >65 years, ability to engage in a simple activity and follow simple directions, ability to understand Taiwanese or Chinese, and presence of problems of social interactions reported by nursing staff ● **Exclusion criteria**: Severe hearing impairment, obvious symptoms, or acute pain or infection	● All participants received group pet robot-assisted therapy twice a week for 4 weeks. Communication and interaction skills were rated using the Assessment of Communication and Interaction Skills (ACIS) score at baseline and at week 4.
Baek et al. [25]	2020	28	● Recruited from hospital settings	● Nonequivalent control group pretest and post-test study design	● **Inclusion criteria**: Korean Mini-Mental Status Examination (MMSE-K) score of 10–19; the ability to read, hear, and communicate; and consent to participate in the study ● **Exclusion criteria**: Neurological or psychological diagnosis other than dementia, or an allergy to dog fur	● Animal-assisted therapy (AAT) group: 14/28 ● Control group: 14/28
Banks and Banks [26]	2002	45	● Long-term health care facilities	● Interventional study	● **Inclusion criteria**: Minimum sixth grade education; ability to speak, read, and write in English; score ≥24 on the MMSE; completion of the Demographic and Pet History Questionnaire; score ≥30 on the UCLA-LS (a score demonstrating a significant degree of loneliness) ● **Exclusion criteria**: Cognitive impairment as stated by a physician, history of psychiatric disorders, or allergies to dogs or cats	● No animal-assisted therapy (AAT): 15/45 ● AAT once/week: 15/45 ● AAT three times/week: 15/45
Takayanagi et al. [27]	2014	30	● Nursing care facility and resident rooms in Japan for elderly patients with dementia	● Observational study	● **Inclusion criteria**: Written informed consent to participate in the study ● **Exclusion criteria**: None	● Two groups; supervised one-on-one interaction with PARO and Stuffed Lion. One session, one session (~15 min) for each intervention per subject, separated by 3–6 months
Bemelmans et al. [28]	2015	71	● Psychogeriatric care institutions for patients with dementia	● Quasi-experimental study	● **Inclusion criteria**: Undesirable psychological or psychosocial unrest or mood based on the professional judgment of the care providers, and care givers experiencing difficulties in providing ADL-care tasks ● **Exclusion criteria**: None	● Supervised one-on-one interaction with PARO or no intervention, two separate phases (crossover) of the study
Moyle et al. [29]	2013	17	● Nursing home for patients with dementia	● Randomized, crossover design	● **Inclusion criteria**: >65, mid- to late-stage dementia or met the criteria per DSM 5 activity ● **Exclusion criteria**: Not blind or severely deaf or physically challenged	● Supervised group interaction with PARO or reading group, three sessions (~45 min)/week for 5 weeks
Valenti et al. [30]	2015	37	● Day care left for patients with dementia	● Interventional study	● **Inclusion and exclusion criteria**: Not specified	● Phase 1: Supervised group therapy (cognitive and physical) with NAO ● Phase 2: Supervised group therapy (cognitive and physical) with PARO; two sessions (30–40 min)/week for 3 months
Lane et al. [31]	2016	23	● Veteran residential care facility for patients with dementia	● Observational study	● **Inclusion and exclusion criteria**: Not specified	● Supervised one-on-one interaction with PARO; three sessions (>5 min) across 1 year
Kramer et al. [32]	2009	18	● Nursing home and participant rooms for patients with dementia	● Interventional study	● **Inclusion criteria**: Able to sit up in a chair or wheelchair, free of visual impairments, and able to move their hands ● **Exclusion criteria**: None	● Supervised one-on-one interaction with AIBO, dog, or no object; one visit (~3 min)/week for 3 weeks
Šabanović et al. [33]	2013	7	● Dementia rehabilitation wing for patients with dementia	● Interventional study	● **Inclusion and exclusion criteria**: Not specified	● Supervised group interaction with Paro. One session/week for 7 weeks
Chu et al. [34]	2009	139	● Residential care facilities for patients with dementia	● Randomized, controlled trial	● **Inclusion and exclusion criteria**: Not specified	● Supervised group interaction across 5 years (2/week)
Kongeable et al. [35]	1989	7	● Patients with dementia	● Observational study	● **Inclusion and exclusion criteria**: Not specified	● Observations in the absence of dog, temporary presence of the dog, and permanent placement of the dog in both settings

AAT—animal assisted intervention.

**Table 2:** Primary and secondary outcomes of studies reviewed.

Article	Primary Outcome	Secondary Outcomes	Limitations
Majic et al. [5]	Reduction in agitation in AAT group (*p* < 0.05)	Reduction in depression in AAT group (*p* < 0.05)	● Small follow-up period
Nordgren and Engström [6]	Physical non-aggressive behaviors decreased over the period, but this was non-significant (*p* > 0.05)	The mean age was 81 years (range 63–91) in the dog assisted intervention group and 83 years (range 71–94) in the control group (*p* = 0.624)	● Small sample size
Liang et al. [7]	Physical aggressive behaviors were reduced in the intervention group, but the decrease was non-significant (*p* > 0.05)	Overall facial expressions improved (*p* > 0.05) but more happiness was observed in participants receiving the intervention (*p* < 0.05); significant social interactions with the intervention included talking with the activity coordinator and staff (*p* < 0.05)	● Small sample size
Olsen et al. [8]	No significant effects on agitation (*p* > 0.05).	No significant effects of the intervention were found from T0 to T1 for depression (*p* > 0.05)	● Not specified
Nurenberg et al. [9]	Agitation scores were significantly reduced in cases of points less than 2 (*p* < 0.05); violent incidences were significantly reduced (*p* < 0.05)	Improved intrusiveness was associated with reduced violence (F = 5.62, df = 1 and 76, *p* = *0*.02) and with a diminished group effect (F = 1.91, df = 3 and 76, ns)	● The groups had been divided into many subgroups given on the type of therapy received which reduced the number of participants per group.
Churchill et al. [10]	Reduced agitation (*p* < 0.05)	Not specified	● Not specified
Richeson et al. [11]	The agitated behaviors of the participants decreased immediately following the intervention phase and increased during the follow-up phase of the pilot study	Social interactions increased significantly from the first week to the last week of the AAT intervention	● Small sample size
Jøranson et al. [12]	Reduction in agitation in PARO versus TAU at 3-month follow-up (*p* < 0.05)	- Reduction in depression in Paro versus TAU at 3-month follow-up (*p* < 0.05) - In those with severe dementia, quality of life scores did not decrease in the PARO group	● Patients were not blinded
Libin and Cohen-Mansfield [13]	Physical agitation and overall agitation decreased with the plush cat (*p* = 0.046, respectively); interactions with the robotic cat also lowered the level of agitation, but it was not significant	Significant increase in pleasure (*p* < 0.01) and interest (*p* < 0.05) scores while playing with plush cat	● Not blinded, small sample size
Sellers et al. [14]	The results indicated a statistically reliable decrease for the total agitated behaviors category (t = 7.05, *p* < 0.0001)	Improvement in social behaviors (*p* < 0.05)	● Small sample size
Moyle et al. [15]	PARO was more effective than the usual care for improving agitation (*p* < 0.05)	● Participants in the PARO group were more verbally engaged than participants in the plush toy group (*p* < 0.05) ● PARO was more effective in improving pleasure (1.12, 95% CI: 1.94–0.29, *p* = 0.008); videos showed that when measured using CMAI-SF, there was no difference between groups	● Small duration for intervention, missing data protocol
Friedmann et al. [16]	Agitation decreased in the PAL group (*p* = 0.423) and remained the same in the reminiscing group (*p* = 0.865)	● Mean age: 79.59 + 9.74 in PAL group vs. 82.11 + 8.36 in the reminiscing group ● Females: 68.2% PAL group vs. 77.8% reminiscing group ● Physical functioning and ADL slightly increased in the PAL group vs. a decrease in the reminiscing group (*p* = 0.306 vs. 0.072) ● PAL group had decreased rates of depression (*p* = 0.013) and there was no change in the reminiscing group (*p* = 0.72)	● Small sample size ● Short follow-up time (3 months)
Zisselman et al. [17]	Reduction in irritable behavior after pet therapy (*p* < 0.07)	● Improved or stable self-care functioning, irritable behavior, and withdrawn behavior in both the intervention and control groups	● Small sample size ● Short follow-up time (5 days) ● Convenience sample ● Per authors, MOSES subscales may have been insensitive to the effects immediately post-intervention (vs. over a longer time frame) ● Exercise (the control activity) has well-documented benefits in older persons
Gustafsson et al. [18]	Less agitated behavior	● Better quality of life	● Small sample size
Krause-Parello and Kolassa [19]	Agitation/delirium not investigated	● Greater decrease in SBP when visited by an animal for those with more poorly rated self-health, higher stress, poorer coping, and men (statistically significant) ● No statistically significant relationship between DBP and any of the variables considered	● Convenience sample; potential selection bias ● Small sample size ● Lack of standardization; variety of dog breeds and handlers
Menna et al. [20]	Agitation/delirium not investigated	● Age: mean + SD: 75 + 6 years ● Range: 62–85 years ● Females: 16 AAT vs. 14 ROT vs. 7 control ● MMSE scores increased by 1.3 in AAT group and by 0.3 in ROT groups (*p* = 0.00) ● Mean GDS scores decreased by 2 in the AAT and 1.1 in the ROT groups (*p* = 0.00) ● No significant changes observed in apathy scores	● Small sample size
Moretti et al. [21]	Agitation/delirium not investigated	● Within the pet therapy group, Geriatric Depression Score (GDS) symptoms decreased by 50% (*p* = 0.013) and MMSE scores increased by 4.5 (*p* = 0.060); the between group comparison showed a positive effect of pet therapy intervention on GDS (*p*= 0.070) ● Most of the participants reported an improvement in their perceived quality of life	● Small sample size ● Short follow-up time (6 weeks) ● Not randomized or double-blinded ● Study design does not allow for separation of the effect of the handler and the effect of the pet
Petersen et al. [22]	Agitation/delirium not investigated	● Increase in RAID (Rating for Anxiety in Dementia), CSDD (Cornell Scale for Depression in Dementia), and GSR (Galvanic Skin Response) scores in the PARO group (*p* = 0.003, 0.001, and 0.0005, respectively) ● Improvements in pulse oximetry and HR in the PARO group (*p* = 0.0001 and 0.0001, respectively)Reductions in pain and behavioral medication doses (*p* = 0.005 and 0.005, respectively)	Short follow-up time (12 weeks)
Song et al. [23]	Agitation/delirium not investigated	● Mean age: 83.94 (SD = 9.29) vs. 85.07 (SD = 6.23) ● Number of female participants: 17 vs. 15 ● Less cognitive deterioration noted in the robotic group (0.06 vs. 0.13, *p* > 0.05) ● Larger decrease in problematic behaviors in the robotic group vs. control (4.47 vs. 1.73, *p* = 0.008) ● Mood and social behavior improved, although this improvement was not statistically significant	● Small sample sizeShort follow-up time (6 weeks)
Sung et al. [24]	Agitation/delirium not investigated	● Improvement in communication and interaction skills post-robot therapy, as measured by increases in Assessment of Communication and Interaction Skills (ACIS) scores at week 4, relative to baseline ● Improvement in activity participation post-robot therapy, as measured by increases in Activity Participation Scale scores at week 4, relative to baseline	● Small sample size ● Short follow-up time (4 weeks) ● Subjective inclusion criteria (i.e., presence of problems of social interactions reported by nursing staff)
Baek et al. [25]	Agitation/delirium not investigated	● No significant differences observed in terms of depression or problematic behaviors ● Cognition improved in AAT group; more improvement was seen at week 8 than at week 4 (*p* < 0.001) ● Mood scores decreased in the AAT group; more improvement was seen at week 8 than week 4 (*p* < 0.014)	● Small sample size ● Post hoc analysis points were not specific ● Response bias; inaccuracy on account of elderly people responding
Banks and Banks [26]	Agitation/delirium not investigated	● AAT significantly reduced loneliness scores (*p* = 0.001), although there was no statistically significant difference between the groups that had AAT once-week vs. three times/week ● A large subpopulation of residents had a strong-life history of owning and caring for pets	● Small sample size ● Study population was self-selected; participation in the study was voluntary and therefore may have been biased towards including individuals that had a preference for interacting with pets
Takanyagi et al. [27]	Not discussed	In both groups: ● More frequent communication with PARO (*p* < 0.05) ● More positive emotional expressivity with PARO (*p* < 0.01) In mild/moderate group only: ● More negative emotions with Lion ● Frequencies of talking to staff member were higher with LionIn severe group only: ● Showed neutral expression more frequently with Lion	● Small sample size ● Certain participants who did not like had limited interactions
Bemelmans et al. [28]	Not discussed	● Therapeutic-related interventions show an increase of IPPA scores by two points (*p* < 0.01)	● Small sample size
Moyle et al. [29]	Not discussed	● The Paro group had pleasure scores, anxiety, and sadness scores following intervention	● Sample size was limited
Valenti et al. [30]	Not discussed	● A significant decrease in the MMSE score in the NAO group, delusions (a significant increase in the NAO group), apathy (a significant decrease in the NAO group), and irritability/lability (a significant increase in the PARO group	● Many patients lost to follow-up
Lane et al. [31]	Not discussed	● Statistically significant increases in positive patient behavioral states when comparing the same group (*p* < 0.05) ● Presence of pre-PARO positive behaviors was significantly associated with post-PARO positive behaviors (*p* < 0.05); the presence of pre-PARO negative behaviors was significantly associated with post-PARO negative behaviors (*p* < 0.05) ● Decreases in negative patient behavioral states (*p* < 0.05)	● Small sample size
Kramer et al. [32]	Not discussed	● All patients exhibited various degrees of interactive behavior	● Small sample size
Šabanović et al. [33]	Not discussed	● PARO increases specific social interactions and activity levels	● Small sample size
Chu et al. [34]	Not discussed	● Increased engagement in the majority of participants	● Not specified
Kongeable et al. [35]	Not discussed	● The presence of the dog increased the number of total social behaviors of the AD clients, but no differences were found in behaviors between its temporary and permanent placement	● Not specified

## Data Availability

Data can be made available upon request to the corresponding author.
